# Comparing two measures of phenological synchrony in a predator–prey interaction: Simpler works better

**DOI:** 10.1111/1365-2656.13143

**Published:** 2019-12-17

**Authors:** Jip J. C. Ramakers, Phillip Gienapp, Marcel E. Visser

**Affiliations:** ^1^ Department of Animal Ecology Netherlands Institute of Ecology (NIOO‐KNAW) Wageningen the Netherlands; ^2^ Biometris Wageningen University & Research Wageningen The Netherlands; ^3^ Michael‐Otto‐Institut im NABU Bergenhusen Germany

**Keywords:** demographic processes, global climate change, match–mismatch hypothesis, *Parus major*, phenology, selection

## Abstract

1. Global climate change has sparked a vast research effort into the demographic and evolutionary consequences of mismatches between consumer and resource phenology. Many studies have used the difference in peak dates to quantify phenological synchrony (match in dates, MD), but this approach has been suggested to be inconclusive, since it does not incorporate the temporal overlap between the phenological distributions (match in overlap, MO).
2. We used 24 years of detailed data on the phenology of a predator–prey system, the great tit (*Parus major*) and the main food for its nestlings, caterpillars, to estimate MD and MO at the population and brood levels. We compared the performance of both metrics on two key demographic parameters: offspring recruitment probability and selection on the timing of reproduction.
3. Although MD and MO correlated quadratically as expected, MD was a better predictor for both offspring recruitment and selection on timing than MO. We argue—and verify through simulations—that this is because quantifying MO has to be based on nontrivial, difficult‐to‐verify assumptions that likely render MO too inaccurate as a proxy for food availability in practice.
4. Our results have important implications for the allocation of research efforts in long‐term population studies in highly seasonal environments.

## Graphical abstract

Phenological mismatch can have important demographic consequences, but the most widely used measure of mismatch by biologists has been argued to be too simplistic. An alternative, theoretically more complete measure has been proposed, but the authors show with empirical data and simulations that this measure is difficult to estimate accurately and hence practically useless. This has important consequences for the collection of resource data for long‐term study populations.

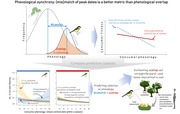

## 1 INTRODUCTION

Organisms in seasonal environments, where the phenology of resource abundance varies from year to year, need to adjust their timing of reproduction to match this variation to ensure successful reproduction (Kokko, 1999; Lepage, Gauthier, & Reed, 1998; Plard et al., 2014; Réale, Berteaux, McAdam, & Boutin, 2003; Reid et al., 2018; Siikamäki, 1998; Smith & Moore, 2005; Verboven & Visser, 1998). Recent decades have seen a growing interest among biologists in the effect of climate warming on changes in phenology (Both et al., 2004; Dunn & Moller, 2014; Durant, Hjermann, Ottersen, & Stenseth, 2007; Parmesan & Yohe, 2003; Plard et al., 2014; Radchuk et al., 2019; Singer & Parmesan, 2010; Visser, 2008; Visser, Noordwijk, Tinbergen, & Lessells, 1998). Typically, warming springs lead to an advancement in phenological events and these advancements occur at different rates between different trophic levels (Kharouba et al., 2018; Thackeray et al., 2010, 2016). The unequal shift in phenology between consumers and their resources, referred to as ‘phenological mismatch’ (Cushing, 1990; Durant et al., 2007; Stenseth & Mysterud, 2002; Visser & Gienapp, 2019), has in some cases been linked to directional selection on consumer phenology (Marrot, Charmantier, Blondel, & Garant, 2018; Reed, Jenouvrier, & Visser, 2013; Visser et al., 1998) and negative effects on consumer demography (Plard et al., 2014).

**Figure 1:**
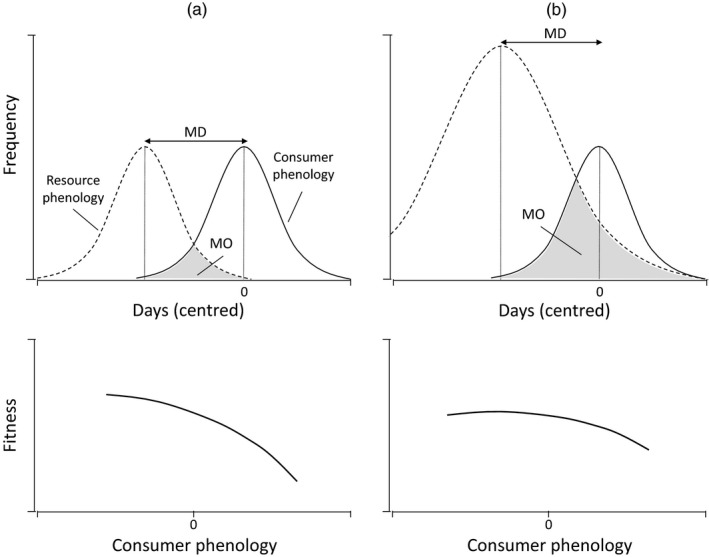
Schematic illustration of the theory behind the two definitions of phenological synchrony (top row; MD = match in peak dates; MO = match in overlap) and its consequences for selection on consumer phenology (bottom row). If the mean phenology of the consumer (solid lines) is out of synchrony with the peak in resource availability (dashed lines; i.e. MD ≠ 0), there will be directional selection on the phenology of the consumer (a). This selection will be less strong under the same degree of MD if the height or the shape of the resource distribution is such that MO is still sizeable (b)

Recently, Lindén (2018) argued that, to better understand the demographic processes mediated by phenological mismatches, a clear and rigorous definition of phenological synchrony is needed. This synchrony between consumer and resource phenology can be described as the difference between the dates when the phenological distributions of the consumer and the resource peak (match in dates, MD). Most studies have used this match in peak dates as a proxy to study phenological synchrony (Kharouba et al., 2018; Reed et al., 2013; Thackeray et al., 2010; Visser et al., 1998). A number of publications (Durant et al., 2005, 2007; Lindén, 2018; Miller‐Rushing, Høye, Inouye, & Post, 2010), however, have suggested that a better measure from the consumer's perspective would be the ‘area of overlap’ under the intersecting distributions of consumer and resource phenology (match in overlap, MO; see Figure 1 for a schematic illustration). The key argument is that resources may be plentiful even when peak dates are out of synchrony when the resource peak is either high (years with plenty of food) or wide (Figure 1b; Lindén, 2018; Miller‐Rushing et al., 2010). Conversely, even if peak dates in phenologies are well matched, overall low resource availability will reduce consumer fitness (Cushing, 1969). Although these two measures of phenological synchrony will often be highly correlated (Lindén, 2018; Miller‐Rushing et al., 2010), it is of interest to test which of them is most relevant for demographic and evolutionary processes.

**Figure 2:**
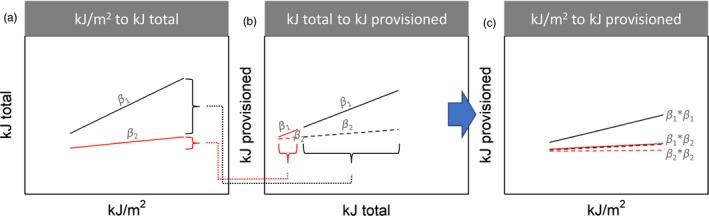
Schematic illustration of the underlying relationship between the amount of food measured on a given day (‘kJ/m^2^’) and the total amount of food effectively consumed or provisioned on that day by the consumer (‘kJ provisioned’). To be able to calculate match in phenological overlap, a correlation (here the regression slopes *β*) must be assumed between (a) the measured and the total available amount of food (‘kJ total’) and between (b) the total amount of food and the amount effectively used by the consumer. Following path analysis, the product of these correlations determines the correlation between kJ/m^2^ and kJ provisioned (c)

One important caveat is that, to be able to calculate the degree of overlap between resource availability and resource requirements, these both need to be expressed in the same units. Food availability is measured in some form of density per spatial unit (perhaps for different resource types if these differ in their quality to the consumer), whereas resource requirements are measured in, for example, energy per day provided to the offspring. The necessary conversion from the former to the latter units requires making important assumptions that are difficult—if not impossible—to verify (see Figure 2 for a schematic illustration). For example, great tits (*Parus major*) are highly dependent on ephemeral abundances of caterpillars (Lepidoptera) to feed their offspring in some regions (Betts, 1955; Lack, 1950; Royama, 1970; Van Balen, 1973). The available caterpillar biomass is expressed in gram/m^2^ per measurement day, as it is based on the amount of caterpillar frass underneath a tree (Visser, Holleman, & Gienapp, 2006). To calculate the overlap in phenology, this measure needs to be converted to the net amount of food available (in kJ) provided by the parents to the individual nestlings on a day. However, this would depend strongly on factors such as the density of the breeding population (through competition) and the spatiotemporal distribution of prey, both in size and numbers, affecting the search time and radius of the parents. Simply quantifying overlap between resource and demand, if possible, assumes that what is available can be effectively used by the consumer, an assumption that may not be true (Figure 2; Pyke, Pulliam, & Charnov, 1977).

MD, on the other hand, is less loaded with such assumptions as it only requires an estimation of the date at which energy requirements are highest (in great tits around day 10 post‐hatching (Keller & Van Noordwijk, 1994; Mols, Van Noordwijk, & Visser, 2005)) and the date at which the biomass of caterpillars is highest. The only assumptions made here are that the critical nestling stage is the same for each brood and that the estimated peak date in biomass is representative for the whole study area, without making assumptions about the absolute height or shape of the temporal distribution. In highly seasonal systems, individuals that are well matched with the peak date in food availability (individual‐level MD) will likely experience the most abundant food conditions and thus have high individual‐level MO, but we will argue in this paper that MD may be nevertheless preferred over MO in such systems based on parsimony and its explanatory power of important demographic processes.

Here, we tested which of the two quantifications of phenological synchrony—the match of peak dates and the phenological overlap—correlated better with selection and offspring recruitment in a wild population of great tits. Great tits in this population depend strongly (albeit not exclusively) on caterpillars (mainly *Operopthera brumata* and *Tortrix viridana*) to raise their offspring (Van Balen, 1973), which are available to them over a span of a few weeks during the breeding season. Egg‐laying date in this population is under increased directional selection due to climate warming, which has been linked to the decreased temporal synchrony with caterpillar abundance (Reed et al., 2013; Visser et al., 2006). We used our long‐term (24 years) data to construct a daily food availability and food requirement profile throughout the breeding season to estimate the overlap between the distributions (MO) as well as the temporal match of peak dates in phenology (MD). Because of the inherent difficulties in estimating food availability (Figure 2), we needed to make strong simplifications as to the daily estimates of food availability. We compared models containing either metric of phenological synchrony to test their importance in predicting (a) the recruitment probability of great tit nestlings and (b) selection on egg‐laying date of the mothers. We additionally conducted simulations to investigate the effect of uncertainty in the estimation of MO on this metric's performance compared to MD in models explaining variation in fitness. We discuss important limitations of constructing food availability and food requirement distributions as well as the appropriateness of using either measure of phenological synchrony to describe ecological interactions between trophic levels.

## 2 MATERIALS AND METHODS

### 2.1 Data collection

We made use of 24 years (1994–2018, excluding 1997; see Section 2.2 for justification) of data on caterpillar availability and great tit breeding data at the Hoge Veluwe National Park (HV; 52°23′N, 05°51′E, central Netherlands). In this area, approximately 400 nest boxes are available for great tits and other hole‐breeding passerines to nest, and the whole reproductive cycle from egg laying to fledging of chicks is monitored. Adults are captured at the nest and identified by means of aluminium leg rings during the chick‐provisioning stage; where possible, earlier identification takes place during the incubation phase. Chicks are ringed and weighed on day 15 post‐hatching, which is close to the date of fledging. For more details on the field procedures and a more accurate description of the study area, see, for example, Reed et al. (2013).

During the breeding season, the caterpillar biomass is estimated by putting up two frass nets (cheese cloths) underneath 15 pedunculate oak (*Quercus robur*) trees spread across the 171‐ha study area (see Visser et al., 2006 for details). These nets capture the droppings (frass) of caterpillars (mostly winter moth *Operopthera brumata* and oak leaf roller *Tortrix viridana*, but some other species are also present) present in the trees. Nets are usually deployed from mid‐April to mid‐June and sampled every 3–4 days. Caterpillar droppings are collected, dried at 60°C for 24 hr and sorted (i.e., debris removed). The dried droppings are then used to calculate the caterpillar biomass whilst correcting for daily temperatures (which affect caterpillar growth) using the equation in Tinbergen and Dietz (1994), which correlates well with biomass obtained from branch samples (Visser et al., 2006). Biomass is first averaged per tree and then across sampling trees to get grams of biomass per square metre for the date that falls in the middle of the sampling days.

In the autumn of each year, beech (*Fagus sylvatica*) crop production in the study area was estimated, since beech nuts constitute an important part of great tit winter diet and influence overwinter survival (Grøtan et al., 2009; Perdeck, Visser, & Van Balen, 2000; Perrins, 1965). Details of the methods are given in Perdeck et al. (2000).

### 2.2 Estimating food availability and food requirement

To estimate caterpillar biomass on a daily basis, we used a smooth spline technique with maximal degrees of freedom to interpolate biomass between measuring days. With this method, biomass outside the measuring period is predicted as a linear function, adopting the slope estimated from the last (or first, depending on the side of the curve) interpolation point. In some years, predicted biomass would therefore linearly decline towards zero. In the majority of years (15 out of 24), however, the slope at the last or first interpolation point (or sometimes both) was slightly positive, leading to an upward prediction of caterpillar biomass at the both ends of the food curve; in these cases, we arbitrarily set biomass beyond the first or last measuring point to zero. We believe this is a reasonable approach, since in most years, the frass sampling scheme started and ended when apparent biomass was (close to) zero. An exception was 1997, where sampling started when caterpillar biomass was clearly on the rise, so we discarded this year from our analyses.

To estimate nestling food requirements, we needed to make a number of estimates. First, we defined brood size of first broods as the number of nestlings present in the nest on day 15 post‐hatching (when they are measured), as much of nestling mortality takes place within the first week (Nur, 1984), likely for reasons other than absolute food shortage (Van Balen, 1973). The number of nestlings present on day 15 is therefore the most accurate representation for days 5 to 15, between which energy requirements are highest (Keller & Van Noordwijk, 1994; Mols et al., 2005; Royama, 1966). We used the observed, age‐specific energy intake as estimated by Mols et al. (2005) and Royama (1966) as a proxy for required energy intake from days 5 to 15 (kJ nestling^−1^ day^–1^; see Figure 1 in Mols et al. (2005)). Note that other factors than age (e.g., ambient temperature, assimilation efficiency) may affect metabolic rates and the required energy intake (Mertens, 1977; O'Connor, 1975; Royama, 1966), but we assumed here that these factors average out in the estimates derived from Mols et al. (2005) and Royama (1966). We divided the required energy intake by the energy content of caterpillars (21.4 kJ/g dry weight (Bell, 1990)) to get the dry biomass of caterpillar required per nestling per day. Assuming 80% wet mass in caterpillars (Bell, 1990), we multiplied the dry biomass by five to get the total required biomass, which amounted to 2.57, 2.92, 3.34, 3.62, 3.90, 3.97, 4.21, 4.37, 4.49, 4.51 and 4.51 g nestling^–1^ day^–1^ from day 5 to 15. This agrees reasonably well with the estimated mean caterpillar intake of 4.66 g nestling^−1^ day^−1^ in great tit broods with nine nestlings found by Gibb and Betts (1963). Daily estimates of food requirements were summed across broods to create a food requirement distribution for all great tit nestlings in the study area. Note, however, that the exclusion of failed broods (those for which we have no measurements on day 15) necessarily disregards the requirements of their nestlings in earlier stages.

One definition of phenological match is the degree of overlap between the food requirement and availability distributions (Durant et al., 2007; Lindén, 2018; Miller‐Rushing et al., 2010). The idea behind it is that even when peak dates differ, mismatch may have little consequences because food is still plentiful (Figure 1). However, food availability and requirements are on a different scale (g/m^2^ vs. g, respectively). We therefore transformed both food availability and requirement to scale between 0 and 1; scaling was done across seasons so as to maintain the original shapes and heights of the distributions as much as possible (Figure S1). Relative overlap (at the population level, MO*_p_*) within a season was then determined using the R package 'sfsmisc' (Maechler, 2017) as an approximation of the integral of the overlapping area (Miller‐Rushing et al., 2010),

$${\text{MO}}_{p}=\frac{\int_{a}^{b}f_{I}\left(t\right)dt}{\int_{c}^{d}f_{F}\left(t\right)dt},$$

where *f_I_*(*t*) and *f_F_*(*t*) represent the functions for the areas under the intersecting (*I*) and the food availability (*F*) curves integrated over time (days) between their respective boundaries *a…b* and *c…d.*

### 2.3 Data analysis: comparing measures of synchrony

We compared the performance of the two main measures of phenological match—that is the temporal synchrony in days between the peak dates of the food needs and the food availability curves (or MD) and the amount of overlap between the food availability and requirement distributions (MO)—in explaining (*a*) offspring recruitment probability and (*b*) the strength of selection on egg‐laying date.

#### 2.3.1 Offspring recruitment probability

We fitted a generalized linear mixed‐effects model (GLMM, package 'lme4'; Bates et al., 2018; Bolker et al., 2009), using maximum likelihood estimation, with a binomial error structure to model nestling recruitment (survival to breed in the next year). We only included broods that did not fail before nestling day 15 (*n* = 14,535 nestlings from 2009 broods, excluding the year 2018 for the lack of recruitment data), since we have the most accurate representation of the number of nestlings in these broods. We are aware that this creates a certain bias in our dataset, but we believe it is acceptable given our aim to compare the relative performance of our metrics of mismatch, rather than to estimate selection. We fitted a ‘base’ model and three different alternative models to assess the relative importance of MD and MO. Here, MD*_b_* (subscript *b* denoting the brood level) is the difference between the date at which the chicks are 10 days old and the peak date in caterpillar biomass, with positive and negative values indicating that the brood was too late or too early, respectively, relative to the peak date in caterpillar biomass. MO*_b_* is a brood‐level proxy for MO, taken as the total amount of food available to a given brood from days 5 to 15, standardized across broods within a season. We expected that recruitment probability would be highest at around MD*_b_* = 0 and to increase with increasing MO*_b_*. The base model consisted of the fixed‐effects breeding pair density (*dens*, the number of breeding pairs in that year) and beech crop index (*BCI*, a three‐level ordinal variable indicating the availability of beech nuts in the autumn following the breeding season) (Grøtan et al., 2009), and the random effects year and brood identity (*brood*) nested within mother identity (*mother*):

$$\text{logit}\left(E{\left[y\right]}_{\mathrm{ijk}}\right)=\alpha_{y}+\beta_{1}{\text{dens}}_{j}+\beta_{2}{\text{BCI}}_{j}+{\text{year}}_{j}+{\text{mother}}_{k}+{\text{brood}}_{l\left(k\right)},$$

where $y_{\mathrm{ijk}}$ is the binary outcome of recruiting or not recruiting, $\alpha_{y}$ is the intercept and $\beta_{n}$ are the slopes associated with each fixed effect. We then fitted alternative models with the following fixed‐effects structure (i) +MD*_b_*; (ii) +MD*_b_* + MD*_b_* ^2^; and (iii) +MO*_b_*. Variance inflation factors (VIF) confirmed that multicollinearity was not an issue in our data (VIF ≤ 1.10). Since we fitted models with similar degrees of freedom, we compared them using Akaike's information criterion corrected for small samples (AIC_c_) to assess whether MD*_b_* outperformed MO*_b_* or vice versa (models within 2 AIC_c_ units from the top‐ranked one were considered competitive; Burnham & Anderson, 2002). To assess the effect sizes of MD*_b_* and MO*_b_*, we obtained the estimates from the most parsimonious model containing the variable of interest and calculated 95% confidence intervals through bootstrapping with 1,000 iterations.

#### 2.3.2 Selection on egg‐laying date

To test the effect of MD and MO on selection on egg‐laying date, we fitted GLMMs where the dependent variable was the number of recruited offspring from a female's brood, assuming a Poisson distribution with a log link (Grøtan et al., 2009). Here, MD*_p_* (subscript *p* denoting the population level) was defined as the population‐mean laying date in that year plus 33 days (see Chevin, Visser, & Tufto, 2015) minus the caterpillar peak date, where negative and positive values of MD*_p_* indicate that the population bred on average too early or too late, respectively, with respect to the peak date of caterpillar biomass. MD*_p_* therefore differs from MD*_b_* in that it is the assumed, rather than the observed, MD. This is because females make the decision to start egg laying approximately a month before nestling demands peak (Visser, Both, & Lambrechts, 2004); some nests may fail well before that time, precisely because females mistimed their reproduction, and this should influence the strength (and direction) of selection on egg laying. For this reason, all known females’ first‐of‐the‐season broods, whether failed or successful, were included in the analysis (*n* = 1764 broods from 1,282 females). MO*_p_* (the population‐level overlap; see Section 2.2) was signed to match the direction of MD*_p_* because it should matter for selection on egg‐laying date whether the overlap was in a positive or negative direction. The ‘base’ model consisted of the fixed‐effects *dens*, clutch size (CS), egg‐laying date (ELD, mean‐centred within years) and BCI (beech crop index), and the random effects year and female identity:

$$\log\left(E{\left[W\right]}_{\mathrm{ij}}\right)=\alpha_{W}+\alpha_{i}+\beta_{1}{\text{dens}}_{j}+\beta_{2}{\text{CS}}_{\mathrm{ij}}+\beta_{3}{\text{ELD}}_{\mathrm{ij}}+\beta_{4}{\text{BCI}}_{j}+{\text{year}}_{j}+e_{\mathrm{ij}},$$

where $W_{\mathrm{ij}}$ is the number of recruited offspring of female *i* in year *j*, $\alpha_{W}$ is the population intercept, $\alpha_{i}$ is the individual female's deviation from the intercept, $\beta_{n}$ are slopes associated with each fixed effect and $e_{\mathrm{ij}}$ is some function of unobserved environmental components. We extended this model using eight variations on MO*_p_* and MD*_p_*. We expected selection on ELD to be negative when the population bred on average too late (MD*_p_* > 0), absent if the population average matched the food peak date (MD*_p_* = 0), and positive if the population bred on average too early (MD*_p_* < 0) (Figure 1). Therefore, we expected an interaction effect between MD*_p_* and ELD on the number of recruits. Similarly, we expected an interaction between signed MO*_p_* and ELD, potentially with an additional quadratic effect since the signed MO*_p_* could theoretically range from –1 to 1, with the lowest fitness expected at MO*_p_* = 0. Lastly, the effect of MD*_p_* may wane when the overall caterpillar peak is high; we therefore tested the effect of the height of the caterpillar peak (HCP). The following alternative models were fitted: (i) + MD*_p_*; (ii) + MD*_p_* + MD*_p_*:ELD; (iii) + MD*_p_* + MD*_p_*:ELD + HCP; (iv) + MO*_p_*; (v) + MO*_p_* + MO*_p_* ^2^; (vi) + MO*_p_* + MO*_p_*:ELD; (vii) + MO*_p_* + MO*_p_*:ELD + HCP (for fair comparison with (iii)); and (viii) + MO*_p_* + MO*_p_*:ELD + MO*_p_* ^2^. Multicollinearity was not an issue in our data (VIF < 2.6). The relative importance of both metrics was judged using AIC_c_ as above. Effect sizes were assessed using the bootstrapped 95% CIs based on 1,000 iterations.

### 2.4 Simulation: Uncertainty in estimating MO*_p_*

Although selection on phenological timing in organisms specialized on highly ephemeral prey should theoretically be largely driven by the amount of temporal overlap (Lindén, 2018; Miller‐Rushing et al., 2010), the effect of true overlap between resource and prey (i.e. what is required and what is effectively available to the consumer) on demographic processes may be difficult to quantify in practice. Two key factors, addressed in simulations here, are (i) the translation from the amount of food measured per unit area to the total availability of food and (ii) the translation from total food availability to the amount that can be effectively consumed or provisioned (see Figure 2).

We simulated data based on 1,500 observations, randomly assigned to one of 23 years (resembling our empirical data). Among‐year variation in MD*_p_* was assumed to be 52, and within‐year variation in phenological timing was assumed to be 22.5, following the Hoge Veluwe great tit population. Each year (*j*) randomly received a MD*_p_* from a normal distribution $\left({\text{MD}}_{p,j}∼N\left(\mu =0,\;\sigma^{2}=52\right)\right)$. We generated a normal density curve (*n* = 1,000) for the food availability for each year, ${\text{food}}_{j}∼N\left(\mu =0,\;\sigma^{2}=140\right)$, which we divided by 30 to get a 30‐day food availability distribution within which densities were averaged. Average density values were then multiplied by 4 divided by the total average to achieve an average of 4 g m^−2^ d^−1^ (as in the Hoge Veluwe data) and subsequently multiplied by 0.2×21.4 to obtain kJ/m^2^ (see Section 2.2). Assuming a 100‐ha study area with ~30% oak trees containing caterpillars (30 ha), we multiplied kJ/m^2^ by $3\times 10^{5}$ to get the total food availability. Individual phenological timing (egg‐laying date, ELD) was drawn as ${\text{ELD}}_{\mathrm{ij}}∼N\left(\mu ={\text{MD}}_{p,j},\;\sigma^{2}=22.5\right)$; note that the time‐lag between laying and the peak in offspring needs (~33 days in great tits) was ignored for simplicity. Food requirement (in kJ) on a given ELD was obtained as $N_{\text{broods}}\times \left[4\;g\;{\text{nestling}}^{-1}\right]\times \left[8\;{\text{nestlings brood}}^{-1}\right]\times \left[0.2\times 21.4\right]$. A smooth spline (with *N*_days_ */*3 degrees of freedom) was applied to the food requirement distribution to remove the ‘sharp edges’. The two distributions were shifted apart using MD*_p_* (i.e. the difference between the dates of the maximum values). Both food requirement and availability were scaled between 0 and 1 across years and MO*_p,j_* was determined by taking the relative integral of the intersecting distributions (see Section 2.2). For simplicity, fitness was assumed to be a quadratic function of MO*_p_* and randomly drawn as $E{\left[W\right]}_{\mathrm{ij}}∼\text{Poisson}\left(\lambda =e^{0.5{\text{MO}}_{p,j}^{2}}\right).$

The context simulated above was based on two basic assumptions concerning food availability: (i) the translation from kJ/m^2^ to total kJ is free of error and (ii) everything available can be effectively used by the consumer (i.e. provisioned to nestlings). We simulated scenarios where the correlation between kJ/m^2^ to total kJ, as well as the correlation between total kJ and ‘provisioned kJ’, was either 1, 0.75, 0.5 or 0.25 (totalling 16 scenarios). The correlation was realized according to the function

$$y=r\sigma_{res_{x,z}}x+{\mathrm{res}}_{x,z}+\sigma_{x}\sqrt{1-r^{2}},$$

where ***y*** and ***x*** are vectors of the new variable and the variable on which the correlation is based, respectively, *r* is the correlation coefficient, ***z*** is a preliminary new variable (z∼N(0,1)), and ${\mathrm{res}}_{x,z}$ is a vector of the residuals of the linear regression between ***x*** and ***z***. We applied a smooth spline (with *N*_days_/3 degrees of freedom) to the resulting food availability (‘provisioned kJ’) before estimating MO*_p_.* We fitted generalized linear models (GLMs) with Poisson errors on the fitness simulated in the ‘ideal’ scenario above. Fixed effects were either MO*_p_* + ${\text{MO}}_{p}^{2}$ (obtained from each scenario) or ${\text{MD}}_{p}\times \text{ELD}$ (since a real quadratic effect of MO*_p_* drives an interaction effect between MD*_p_* and ELD); the two models were compared using AIC_c_. The entire procedure was iterated 1,000 times.

## 3 RESULTS

### 3.1 Association between population‐level MD and MO

The proportional phenological overlap between the food availability and food requirement distributions at the population level (MO*_p_*; Figure S1) correlated nonlinearly with the match in peak dates in phenologies (MD*_p_*) (Figure S2; coefficients [bootstrapped 95% CI] of a beta‐regression model: MD*_p_*: –0.039 [–0.142, 0.133]; MD*_p_* ^2^: –0.011 [–0.029, –0.001]; pseudo‐*r* ^2^ = 0.36 [0.08, 0.63]). That is, the temporal proportional overlap between food requirements and availability was largest in years when the date of the peak requirements was well matched with the date of peak caterpillar availability, although the confidence interval widened at the lowest values of MD*_p_*. We may therefore predict that MD and MO drive offspring recruitment and selection on breeding time to a similar degree.

### 3.2 Relative performance of MD and MO in explaining offspring recruitment and selection on egg‐laying date

**Figure 3:**
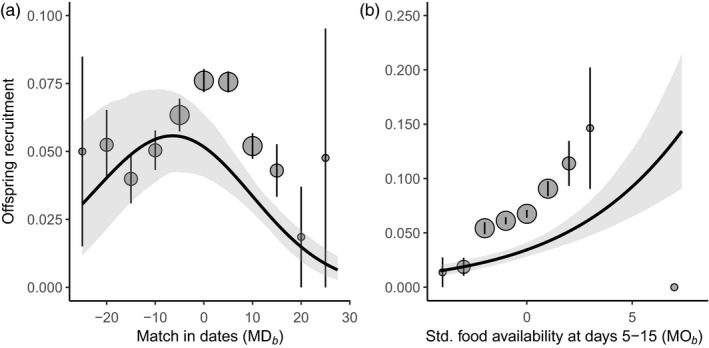
Great tit nestling survival to the next breeding season as a function of (a) MD*_b_* (i.e. the date on which nestlings are 10 days old minus the peak date of caterpillar biomass) and (b) MO*_b_* (i.e. food availability to 5‐ to 15‐d‐old nestlings, standardized across broods within a season). Points are binned raw means with their standard errors, plotted for visual purposes only, with symbol sizes corresponding to sample sizes (small: ≤ 100 nestlings; medium: < 100 and ≤ 1,000 nestlings; large:> 1,000 nestlings). The prediction lines and 95% bootstrapped CIs (shadings) were derived from the 3rd (a) and 4th (b) models in Table 1a, estimated for intermediate *BCI* and keeping breeding density at its mean. Note the different scaling on the y‐axes

The best GLMM explaining variation in offspring recruitment probability contained MD*_b_*, including its quadratic term, but not MO*_b_* (Table 1a). Offspring recruitment was highest when broods with 10‐d‐old nestlings were close to matching with the peak date of caterpillar availability (Figure 3a; estimate MD*_b_* [bootstrapped 95% CI]: –0.025 [–0.039, –0.011]; MD*_b_* ^2^: –0.002 [–0.003, –0.001]; see also Visser et al. (2006); see Table S2a for further estimates from the top‐ranked model). Recruitment probability correlated significantly positively with MO*_b_* in a model that did not contain MD*_b_* (Figure 3b; 0.208 [0.150, 0.273]), but this model performed worse than the best model that contained MD*_b_* and MD*_b_* ^2^ (ΔAIC_c_ = 3.79).

**Table 1:** Comparison of models containing the two metrics of phenological synchrony (MD and MO) explaining variation in (a) great tit nestling survival to recruitment (GLMMs, binomial error; *n* = 14,535 nestlings from 2009 broods) and in (b) number of recruited offspring (selection for great tit egg‐laying date; GLMMs, Poisson error; *n* = 1,764 broods from 1,282 females)

Model terms	ΔAIC_c_
*(a) Offspring recruitment probability*	
Dens + BCI	33.76
Dens + BCI + MD*_b_*	10.59
Dens + BCI + MD*_b_* + MD*_b_* ^2^	0
Dens + BCI + MO*_b_*	3.79
*(b) Selection on timing*	
Dens + CS + ELD + BCI	6.28
Dens + CS + ELD + BCI + MD*_p_*	7.95
Dens + CS + ELD + BCI + MD*_p_* + MD*_p_*:ELD	0
Dens + CS + ELD + BCI + MD*_p_* + MD*_p_*:ELD + HCP	1.00
Dens + CS + ELD + BCI + MO*_p_*	6.61
Dens + CS + ELD + BCI + MO*_p_* + MO*_p_* ^2^	8.32
Dens + CS + ELD + BCI + MO*_p_* + MO*_p_*:ELD	5.73
Dens + CS + ELD + BCI + MO*_p_* + MO*_p_*:ELD + HCP	6.73
Dens + CS + ELD + BCI + MO*_p_* + MO*_p_*:ELD + MO*_p_* ^2^	7.44

Note

dens = breeding pair density; BCI = beach crop index; MD*_b_* = brood‐level phenological match in dates; MO*_b_* = standardized food availability to a nest (days 5–15), as a proxy for brood‐level match in overlap; CS = clutch size, ELD = egg‐laying date (centred within years); MD*_p_*: population‐level phenological match in dates; MO*_p_* = population‐level phenological match in overlap; HCP: height of the caterpillar peak. Random effects were (*a*) year, mother and brood identity (nested within mother), and (*b*) year and female identity.

**Figure 4:**
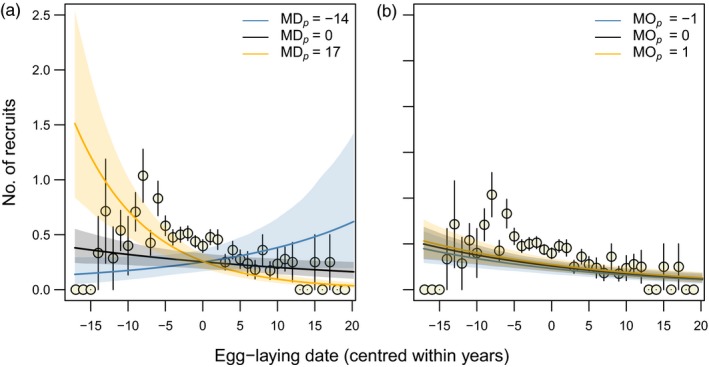
Number of recruited great tit offspring as a function of centred egg‐laying date interacting with (a) MD*_p_* (0 = perfect match) and (b) MO*_p_* (signed to match the direction of MD*_p_*). Data points are means ± SE of raw data binned per centred laying date. Lines and shadings are estimates and bootstrapped 95% CIs from GLMMs with Poisson errors, fitted for three scenarios of MD*_p_* and MO*_p_* (estimates from the 3rd (a) and 7th (b) models in Table 1b, for intermediate *BCI* and with other parameters kept at their means)

Since food availability determines offspring recruitment probability (see above; e.g. Durant et al., 2005; Reed et al., 2013; Toupoint et al., 2012), reproductive success should decline with breeding time if the population breeds on average too late in relation to caterpillar phenology and increase if it breeds to early, indicating selection for earlier and later breeding, respectively. The best model explaining variation in the number of recruited offspring contained the interaction MD*_p_* × ELD but not MO*_p_* × ELD (ΔAIC_c_ = 5.73) or any other combination with MO*_p_* (Table 1b; ELD: –0.023 [–0.043, –0.003]; MD*_p_*: 0.001 [–0.012, 0.014]; MO*_p_*: 0.086 [–0.028, 0.213]; ELD × MD*_p_*: –0.004 [–0.007, –0.002]; ELD × MO*_p_*: –0.024 [–0.044, 0.004]; see Table S2b for further estimates from the top‐ranked model). The predicted number of recruits declined with ELD in years with strong positive MD*_p_* and increased in years with strong negative MD*_p_* (Figure 4a), whereas this effect was virtually absent for MO*_p_* (Figure 4b). The inclusion of *HCP* (peak height) did not improve the model fit (Table 1b; 0.005 [–0.001, 0.011]; ΔAIC_c_ = 1.00). In an additional set of analyses (Supporting Information S1), we replaced HCP with a measure of the skewness or kurtosis of the food availability distribution, but neither contributed to a better fit of the MO*_p_* model, although replacing HCP with skewness led to a better fit in the ELD × MD*_p_* model (ΔAIC_c_ = 2.49; Table S1).

### 3.3 Simulation results

**Figure 5:**
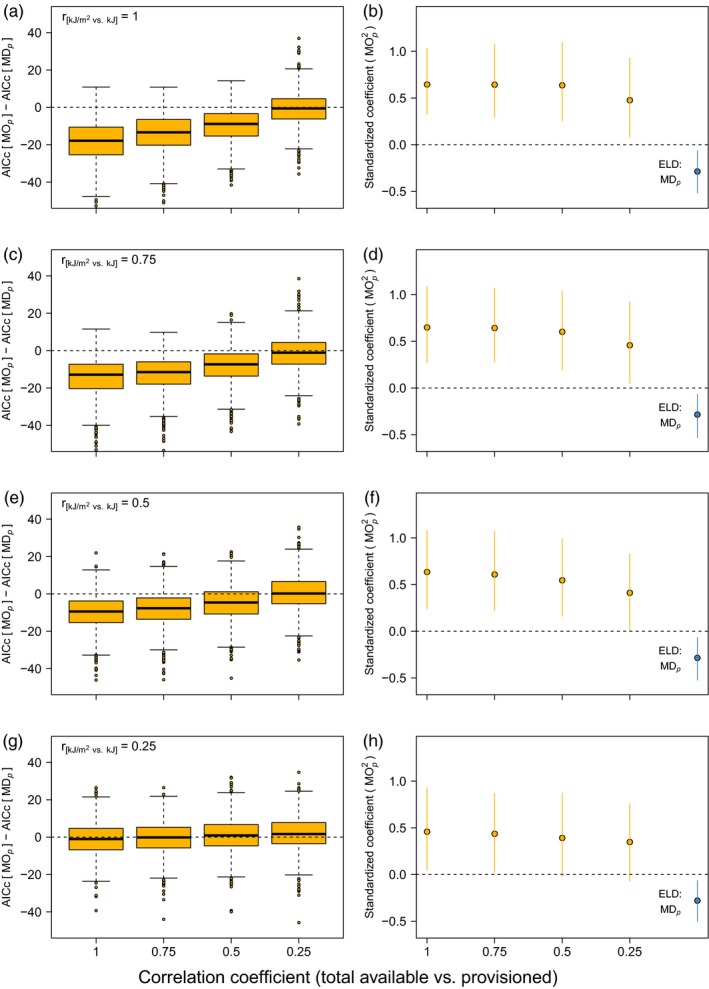
Results of the simulations exploring the effect of uncertainty in the match in overlap (MO*_p_*) due to uncertainties in the translation from kJ/m^2^ to total kJ available (top to bottom panels) and from total kJ available to kJ provisioned (horizontal axis). Shown are the differences between the performance of GLMs on fitness (simulated as a quadratic function of MO*_p_*) with either ${\text{MO}}_{p}+{\text{MO}}_{p}^{2}$ or ELD × MD*_p_* as fixed effects (a, c, e, g) and the standardized coefficients (+95% CI) of ${\text{MO}}_{p}^{2}$ and ELD:MD*_p_* (b, d, f, h). Negative ΔAIC_c_ values indicate a better fit of MO*_p_* model, whereas positive values indicate better fit of the MD*_p_* model

The degree of uncertainty in the translation from (i) food availability per unit area (kJ/m^2^) to the total availability (total kJ) and (ii) total kJ to effective availability (kJ provisioned) interactively determined the performance and effect sizes in the fitness GLMs (Figure 5). Fitness, which was simulated as a squared function of MO*_p_* assuming perfect correlations, was best modelled by ${\text{MO}}_{p}$ and ${\text{MO}}_{p}^{2}$ when the correlation between kJ/m^2^ and total kJ as well as that between total and provisioned kJ was accurate (Figure 5a, b). However, as the correlation between total and provisioned kJ decreased in strength, ΔAIC_c_ values increased (in favour of the ELD × MD*_p_* model) and (standardized) coefficients of ${\text{MO}}_{p}^{2}$ decreased (Figure 5, horizontal axes). This effect was exacerbated as the correlation between kJ/m^2^ and total kJ decreased (Figure 5, top to bottom panels), and in the scenarios of highest uncertainty the MD*_p_* model performed at least equally well or slightly better than the MO*_p_* model. Thus, uncertainty in how ‘measured’ food availability translated into total availability and, subsequently, into effective usage of this availability by the consumer rendered MO*_p_* a less‐than‐ideal explanatory variable for variation in fitness.

## 4 DISCUSSION

Our empirical results show that the phenological synchrony of food availability and food requirements in our population can be better estimated as the difference in days between the mean phenology (MD) than as the relative degree of overlap of these two distributions (MO), even though MD and MO correlated with one another in a predictable fashion, both at the population level (Figure S2) and the brood level (through nest‐level food availability; Figure S3). At the brood level, more food available during critical nestling stages increased survival probability of offspring, but this metric was statistically outperformed by a simple measure of the brood's match with the peak date in caterpillar availability. Similarly, at the population level, females’ reproductive timing (ELD) interacted more significantly with MD*_p_* than with MO*_p_* to predict the number of surviving offspring, indicating that selection was driven by a temporal mismatch with the food peak (see Ramakers, Gienapp, & Visser, 2018). In the latter analysis, the estimate of the main effect MD*_p_* (and that of MO*_p_*, for that matter) was small, with confidence intervals largely overlapping zero, confirming previous findings for this population that phenological mismatch does not in and of itself affect the mean fitness in the population (Reed et al., 2013).

Intuitively, there are two (interrelated) ways in which we can interpret the results, the first one being mainly methodological and the second one more biological in nature. First, our estimates of daily food requirements or availability (or both) may be inaccurate, thus preventing us from reliably estimating phenological overlap. Our simulations confirmed that error in the estimation of MO*_p_* can diminish the power of this metric in predicting the number of recruits. Getting accurate estimates of phenological overlap between predator and prey (Lindén, 2018) requires sufficient knowledge of resource availability (e.g. total number of prey, their size and their spatiotemporal distribution) but this will be challenging in natural systems for various reasons. For example, to construct the food abundance throughout the entire breeding season, we needed to extrapolate the shape of the distribution outside the measuring period, when the values at either the first or the last measurement were >0 (see Section 2 for how these data were treated). Similarly, to construct a food requirement distribution, we had to make assumptions about age‐specific energy requirements and food intake rates in great tit nestlings, which may vary with context (Mertens, 1977; O'Connor, 1975; Royama, 1966). Even if we assumed that we managed to estimate both distributions with reasonable accuracy, we had to transform them both to get them on the same scale. This means that our measure of MO was now not an absolute measure of overlap (MO*_p_*), which has been argued to matter most in consumer–resource interactions (Durant et al., 2007; Lindén, 2018; Miller‐Rushing et al., 2010). In our analysis of offspring recruitment, we standardized food availability across broods such that it became a measure of what was available relative to other broods in that year, likely rendering MO*_b_* a more suitable measure of overlap than MO*_p_* (Figure 3b). More problematically, however, we needed to make nontrivial assumptions about the translation from the amount of food sampled to the total amount available in the study area and how this food is subsequently used by the consumer (see below). This, as shown by our simulations, poses problems for the estimation of MO as a proxy for phenological synchrony.

The second, biological reason why MD may have outperformed MO in our analyses lies in the nature of interaction between predator and prey. Even if we assumed we had an accurate estimate of food availability and requirements and thus an accurate measure of overlap (e.g. Figure 5a), the amount of food effectively available to great tit nestlings would depend strongly on a combination of factors such as spatiotemporal distributions and aggregations of caterpillars, population densities (affecting competition) and foraging radii (affecting e.g. the probability to detect prey). If a given breeding pair has access to one particular tree that is teeming with caterpillars, all the food available in the remainder of the study area becomes irrelevant (Naef‐Daenzer & Keller, 1999). In great tits, the link between prey density and prey‐encounter rate has been experimentally demonstrated to be far from straightforward. Mols et al. (2004) found that experimentally doubling the caterpillar density in a tree increased the encounter rate by 72% and not by 100%—a result expected from functional response theory (Denny, 2014; Hastings, 1997). Interestingly, however, previous removal of caterpillars by other great tits further impaired the probability to detect the remaining prey (Mols et al., 2004), possibly because the remaining caterpillars responded to the previous encounter by hiding or because they represented a non‐random subset of caterpillars that were difficult to find in the first place (Charnov, Orians, & Hyatt, 1976). These findings suggest that even a ‘highly accurate’ estimate of MO (i.e. based on what is strictly available and what is required) may still be uninformative for the demographic processes we wish to study. Although MO is often inherently related to MD, the latter measure does not make assumptions about temporal fluctuations in food availability and requirement and how these factors interact, which in reality will be very difficult to quantify. We therefore argue that MD in our system is a more parsimonious and hence more useful quantification of phenological synchrony than MO.

Our empirical findings echo previous work that highlight match of peak dates in phenology as an important factor influencing mother and offspring fitness (Reed et al., 2013; Vatka, Orell, & RytkÖnen, 2011). Naturally, this will not necessarily be true in all study systems: in species that are not highly dependent on a single food type, or whose food does not exhibit a well‐defined seasonal distribution, demographic processes will either depend more strongly on MO or on neither MD nor MO (Dunn, Winkler, Whittingham, Hannon, & Robertson, 2011; Durant et al., 2005). However, studies reporting fitness and demographic consequences in this context so far have generally used (proxies of) MD to quantify phenological mismatch and reported reduced fitness in years when temporal mismatch was high (Arlt & Pärt, 2017; Marrot et al., 2018; Plard et al., 2014; Regular et al., 2014). Durant et al. (2005), on the other hand, quantified effects of MD and food abundance on population indices of reproductive success in three study systems and found that in two of them food abundance was a better predictor than MD. In one of these two systems (Soay sheep *Ovis aries*), however, food (i.e. vegetation, indicated by integrated NDVI) was only weakly seasonal, whereas in the other system (Atlantic puffins *Fratercula arctica* and herring *Clupea harengus*), an incomplete measure of fitness (i.e. the number of fledged chicks) was used (Durant et al., 2005), making these studies not totally comparable to ours. There is hence yet no a priori expectation that consumer–prey interactions in other highly seasonal environments should be critically different from that of the great tits reported here.

Lindén (2018) makes the recommendation that instead of focussing solely on phenological synchrony (e.g. of peak dates) to describe ecological interactions between trophic levels, we may wish to also incorporate information on abundances across the season. Whilst we agree with the underlying logic, we have shown that phenological match in peak dates (MD) is in fact a reasonable proxy describing demographic processes in a system in which the consumer is strongly dependent on highly ephemeral prey whose effective availability may nevertheless be difficult to quantify accurately. In some cases, adding some measurable feature of the food distribution (the maximum height, or a measure of skew; Table S1) to fitness/selection models that include MD may improve the fit of these models (see Vatka, Rytkonen, & Orell, 2014; Visser et al., 2006), and this may in practice be the closest approximation to incorporating a measure of overlap. An absolute measure of overlap as proposed by Lindén (2018), however, will be difficult because of imperfect knowledge of the underlying relationships between what is measured and what is used by the consumer. In addition, in estimating selection on consumer phenology, MO*_p_* needs to be signed to match the direction of MD*_p_* (since selection is directional and hence relative to a reference date), suggesting that MO*_p_* does not readily lend itself for statistical estimation of selection. These principles may apply to any symbiotic interaction between two species (e.g. plant–pollinator or parasite–host). The important advantage of using MD to quantify phenological synchrony is that it requires a comparatively straightforward way of collecting data that, in any case, will be more parsimonious and perhaps more accurate than any approximation of absolute resource availability throughout the season. This is because MD ‘only’ requires sampling the resource (e.g. per unit area) at regular time intervals, preferably across multiple sites within the study area, spanning a wide‐enough range to be able to estimate when abundance peaks. As we have shown here with our great tit and caterpillar data, we can attempt to develop proxies of phenological overlap (MO) but our expectation is that in many contexts, MD will be a more effective and less biased measure of phenological synchrony, as also illustrated by our simulations (Figure 5).

We would encourage other researchers of long‐term population studies of species highly dependent on an ephemeral resource to think critically about how the best data necessary for quantifying phenological synchrony can be collected. It is these long‐term data that will enable us to understand the long‐term population consequences of phenological mismatch under a changing environment (Clutton‐Brock & Sheldon, 2010; Visser, 2008).

## AUTHORS' CONTRIBUTIONS

J.J.C.R., P.G. and M.E.V. designed the study; M.E.V. collected the frass samples; J.J.C.R. analysed the data and drafted the manuscript; and P.G. and M.E.V. commented on and helped revising the manuscript.

## Data Availability

The data and an R script for the simulation are available on Dryad Digital Repository: https://doi.org/10.5061/dryad.q573n5tf1 (Ramakers, Gienapp, & Visser, 2019).
