# Supplementary material for: Comparing two measures of phenological synchrony in a predator–prey interaction: Simpler works better
Source: J Anim Ecol. 2019 Dec 17;89(3):745–56. doi: 10.1111/1365-2656.13143 (PMC7078916; doi:10.1111/1365-2656.13143)
Supplement: Supplementary file 1 [file JANE-89-745-s001.docx]

**Supplementary Info S1**

In testing which of the match in overlap (*MO_p_*) and match in dates (*MD_p_*) performs better in explaining selection on egg-laying date (see Methods, main text), we additionally replaced the height of the caterpillar peak (*HCP*, a parameter describing a feature of the original food distribution) with a measure of (1) skewness and (2) kurtosis. The rationale was that the precision with which we can estimate the overlap between food requirements and food availability may be impacted by the shape of the food-availability distribution. Also, the estimation of the effect of *MD_b_* on selection may be improved when we include a feature of the food distribution. The same should obviously be true for the food-requirements distribution but this distribution does not vary substantially between years (see Figure S1).

To determine skew and kurtosis in the food-availability distribution, we changed it into a sham frequency distribution by multiplying the estimated daily food availability by 1000. We then used the ‘e1071’ package (Meyer et al. 2015) to calculate kurtosis and skewness. We performed a comparative analysis (using AIC corrected for small sample sizes) of the GLMMs as described in the main Methods but replaced *HCP* (in two models) by either skewness or kurtosis.

Neither skewness nor kurtosis changed the relative performance of models containing either mismatch metric explaining variation in the number of recruited offspring (Table S1), although the addition of skewness improved the fit of the model that contained the interaction between *ELD* and *MD_p_.*

**Table S1**. Comparison of GLMMs containing the two metrics of phenological synchrony at the population level (*MD_p_* and *MO_p_*) explaining variation in the number of recruited offspring *P. major*.

| Model terms | ΔAIC_c_ |
| --- | --- |
| Dens + CS + ELD + BCI | 8.76 |
| Dens + CS + ELD + BCI + MD_p_ | 10.44 |
| Dens + CS + ELD + BCI + MD_p_ + MD_p_:ELD | 2.49 |
| Dens + CS + ELD + BCI + MD_p_ + MD_p_:ELD + kurt | 2.14 |
| Dens + CS + ELD + BCI + MD_p_ + MD_p_:ELD + skew | 0 |
| Dens + CS + ELD + BCI + MO_p_ | 9.10 |
| Dens + CS + ELD + BCI + MO_p_ + MO_p_^2^ | 10.81 |
| Dens + CS + ELD + BCI + MO_p_ + MO_p_:ELD | 8.22 |
| Dens + CS + ELD + BCI + MO_p_ + MO_p_:ELD + kurt | 8.86 |
| Dens + CS + ELD + BCI + MO_p_ + MO_p_:ELD + Skew | 8.37 |
| Dens + CS + ELD + BCI + MO_p_ + MO_p_:ELD + MO_p_^2^ | 9.92 |

Note. dens = breeding-pair density; *BCI* = beach crop index; *CS* = clutch size, *ELD* = egg-laying date (centred within years); *MD_p_*: population-level phenological match in dates; *MO_p_* = population-level phenological match in overlap; *kurt* = kurtosis of caterpillar distribution; *skew* = skewness of caterpillar distribution. Random effects were year and female identity.

*Reference used:*

Meyer, D., E. Dimitriadou, K. Hornik, A. Weingessel, F. Leisch, C.-C. Chang, C.-C. Lin, and M. D. Meyer. 2015. Package ‘e1071’. The Comprehensive R Archive Network. Available at: <https://cran.r-project.org/web/packages/e1071/e1071.pdf>.

**Table S2**

Estimated coefficients from top-ranked GLMMs explaining variation in (*a*) offspring recruitment probability and (*b*) selection on egg-laying date in Dutch great tits (see main text for details).

| Model term | coefficient (95% CI_boot_) |
| --- | --- |
| *(a) Offspring recruitment probability* | |
| Intercept | –2.501 (–2.999, –2.006) |
| Breeding density | –1.166 (–1.741, 0.607) |
| BCI-1 | - |
| BCI-2 | 0.512 (0.276, 0.748) |
| BCI-3 | 1.184 (0.999, 1.379) |
| MD_b_ | –0.025 (–0.039, –0.011) |
| MD_b_^2^ | –0.002 (–0.003, –0.001) |
| Random (variance): |  |
| Brood ID : Mother ID | 0.246 |
| Mother ID | 0.057 |
| Year | 0.101 |
|  |  |
| *(b) Selection on timing* | |
| Intercept | –1.719 (–2.435, –1.050) |
| Breeding density | –0.932 (–1.578, –0.281) |
| Clutch size | 0.089 (0.050, 0.130) |
| Egg-laying date (centred) | –0.023 (–0.043, –0.003) |
| BCI-1 | - |
| BCI-2 | 0.296 (0.007, 0.581) |
| BCI-3 | 1.068 (0.822, 1.321) |
| MD_p_ | 0.001 (–0.012, 0.014) |
| Egg-laying date : MD_p_ | –0.004 (–0.007, –0.002) |
| Random (variance): |  |
| Year | 0.213 |
| Female ID | 0.042 |

**Figure S1**

**
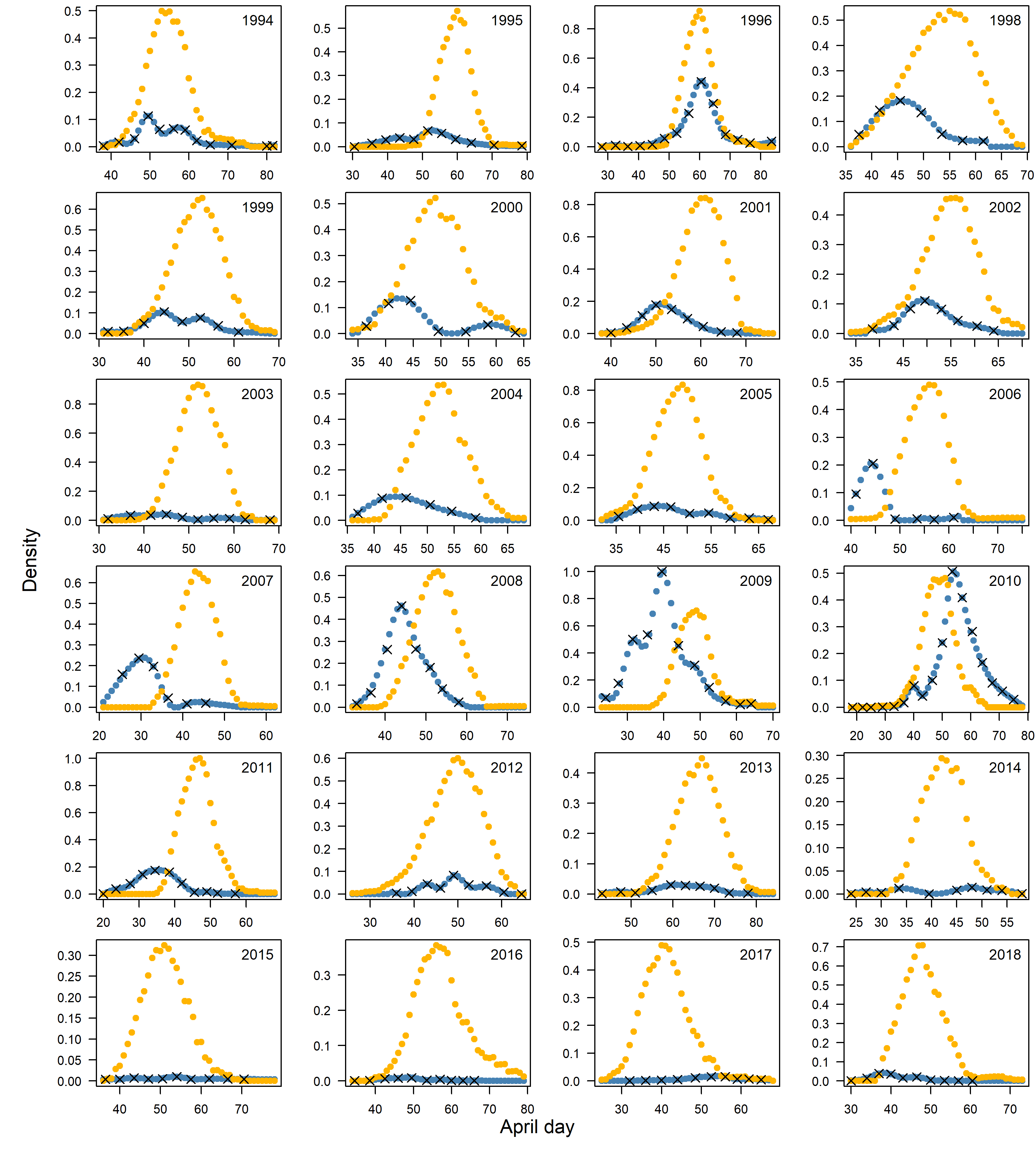
Figure S1**. Availability of caterpillars (blue dots) and food requirements of *Parus major* nestlings (orange dots) throughout the breeding season in 24 years at the Hoge Veluwe National Park. Values of both curves are scaled between 0 and 1 across all years (see main text for details). Black crosses indicate actual caterpillar sampling days.

**Figure S2**

**
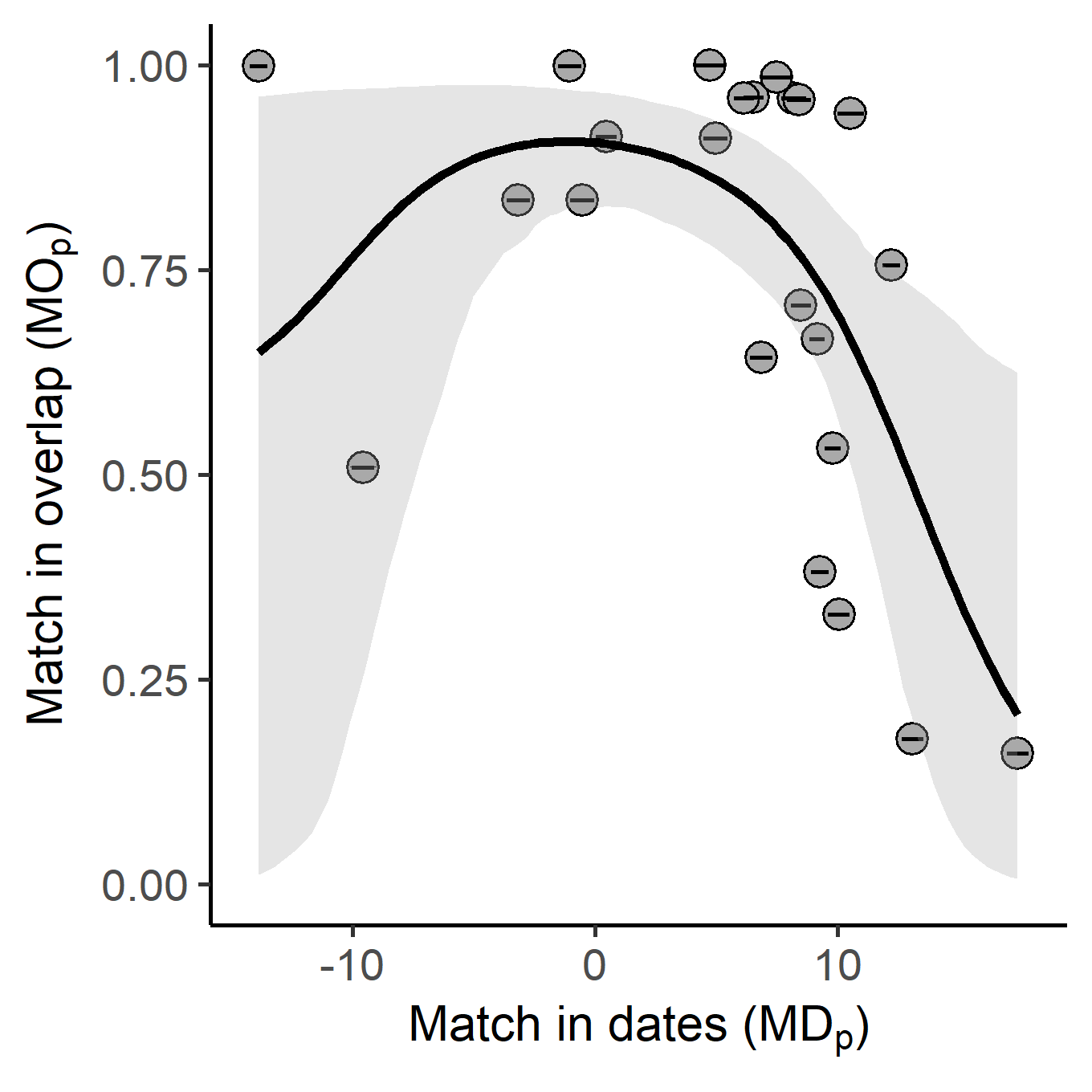
**

**Figure S2.** Coefficient of match in overlap (*MO_p_*) against the match in dates (*MD_p_*) in phenology at the population level (denoted by subscript *p*) in great tits. *MO_p_* is the proportion of the food requirement distribution overlapping with the food availability distribution (see main text for details). *MD_p_* is the difference between the average egg-laying date + 33 days and the peak date of caterpillar biomass, where positive values indicate that the population on average bred too late relative to the food peak, and negative values indicate that it bred too early (0 = perfect match). Line and shading represent estimates and 95% bootstrapped CI from a beta-regression model, accounting for standard errors in *MD_p_* (horizontal lines).

**Figure S3**


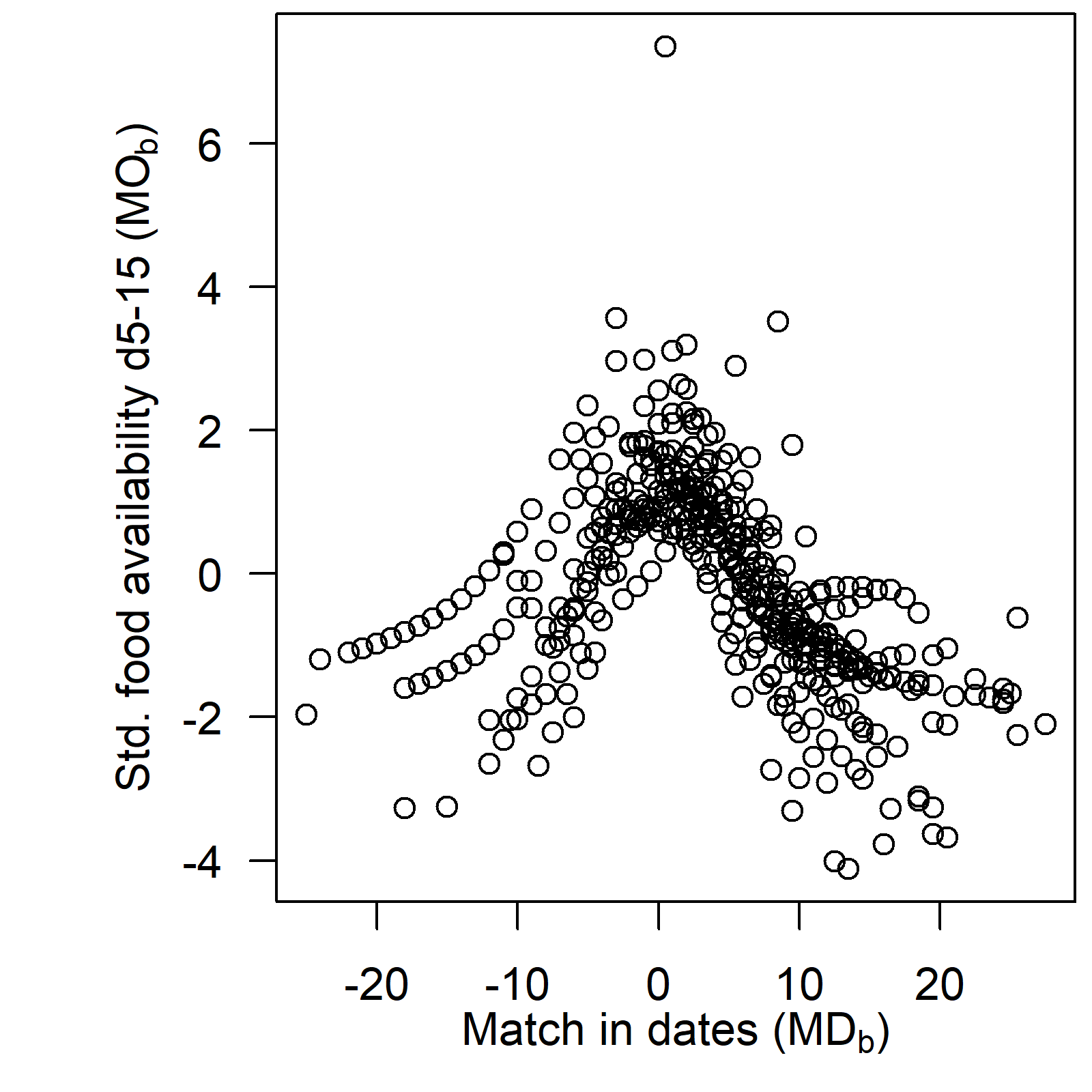


**Figure S3.** Brood-level, within-season-standardized food availability from nestling day 5 to 15 (*MO_b_*) plotted against the brood-level match in peak dates (caterpillar peak date – date at chick age 10d). Broods that were well matched with the food peak (*MD_b_* = 0) had access to most food relative to other broods that were either too early (*MD_b_* < 0) or too late (*MD_b_* > 0).
